# Cryo-EM structure of cannabinoid receptor CB1-β-arrestin complex

**DOI:** 10.1093/procel/pwad055

**Published:** 2023-12-15

**Authors:** Yuxia Wang, Lijie Wu, Tian Wang, Junlin Liu, Fei Li, Longquan Jiang, Zhongbo Fan, Yanan Yu, Na Chen, Qianqian Sun, Qiwen Tan, Tian Hua, Zhi-Jie Liu

**Affiliations:** iHuman Institute, ShanghaiTech University, Shanghai 201210, China; iHuman Institute, ShanghaiTech University, Shanghai 201210, China; iHuman Institute, ShanghaiTech University, Shanghai 201210, China; School of Life Science and Technology, ShanghaiTech University, Shanghai 201210, China; iHuman Institute, ShanghaiTech University, Shanghai 201210, China; iHuman Institute, ShanghaiTech University, Shanghai 201210, China; iHuman Institute, ShanghaiTech University, Shanghai 201210, China; School of Life Science and Technology, ShanghaiTech University, Shanghai 201210, China; iHuman Institute, ShanghaiTech University, Shanghai 201210, China; School of Life Science and Technology, ShanghaiTech University, Shanghai 201210, China; iHuman Institute, ShanghaiTech University, Shanghai 201210, China; School of Life Science and Technology, ShanghaiTech University, Shanghai 201210, China; iHuman Institute, ShanghaiTech University, Shanghai 201210, China; iHuman Institute, ShanghaiTech University, Shanghai 201210, China; iHuman Institute, ShanghaiTech University, Shanghai 201210, China; iHuman Institute, ShanghaiTech University, Shanghai 201210, China; School of Life Science and Technology, ShanghaiTech University, Shanghai 201210, China; iHuman Institute, ShanghaiTech University, Shanghai 201210, China; School of Life Science and Technology, ShanghaiTech University, Shanghai 201210, China; Institute of Molecular and Clinical Medicine, Kunming Medical University, Kunming 650500, China

## Dear Editor,

G protein-coupled receptors (GPCRs) play a vital role in regulating almost every aspect of human physiology, making up more than one-third of marketed drug targets (Santos et al., 2017). GPCRs orchestrate their signalling through interactions with three distinct downstream protein families: G proteins, G protein-coupled receptor kinases (GRKs), and arrestins (Santos et al., 2017). While G protein-mediated signalling is initiated upon GPCR stimulation, activated GPCRs return to their basal levels through a GRK- and arrestin-regulated desensitization process (Santos et al., 2017). In addition to modulating receptor desensitization, β-arrestin also regulates downstream events that are distinct from classical G protein signalling (Ahn et al., 2020). Although a number of GPCR-G protein complex structures were obtained, only a few GPCR-β-arrestin complex structures have been determined. In addition, most of the solved receptor-β-arrestin complexes were stabilized by chemical crosslinking (Huang et al., 2020), *in vitro* phosphorylation (Lee et al., 2020; Staus et al., 2020), *in vitro* binding (Bous et al., 2022), or directly fused the β-arrestin to the receptor (Cao et al., 2022), thus the more physiological relevant GPCR-β-arrestin complex structures are needed.

Attempts to identify biased ligands have persisted for many GPCRs, despite, in many cases, no clear indication yet of which pathways might mediate the therapeutic benefits (Patel et al., 2021). One such example is the type-1 cannabinoid receptor (CB1), which is one of the most abundant GPCRs in the central nervous system. It mediates complex pharmacological processes and is an important therapeutic target for treating many neurological disorders (Patel et al., 2021). However, the development of successful therapeutics has been hampered by unwanted side effects, and β-arrestin biased signalling of CB1 has been shown to deliver potential required effects and thus is currently the subject of intense drug discovery exploration (Liu et al., 2021).

To gain a comprehensive insight into CB1 downstream signalling mechanism at the molecular level, we solved the cryo-electron microscopy (cryo-EM) structure of AM841-bound CB1 in complex with β-arrestin-1 (βarr1) assembled by co-expression with G protein-coupled receptor kinase 3 (GRK3). Combining with the previously determined CB1 structures in inactive (Hua et al., 2016), active-like (Hua et al., 2017), and active (Hua et al., 2020) states, our work presents a more complete structural framework of CB1-mediated signalling.

**Figure 1.**
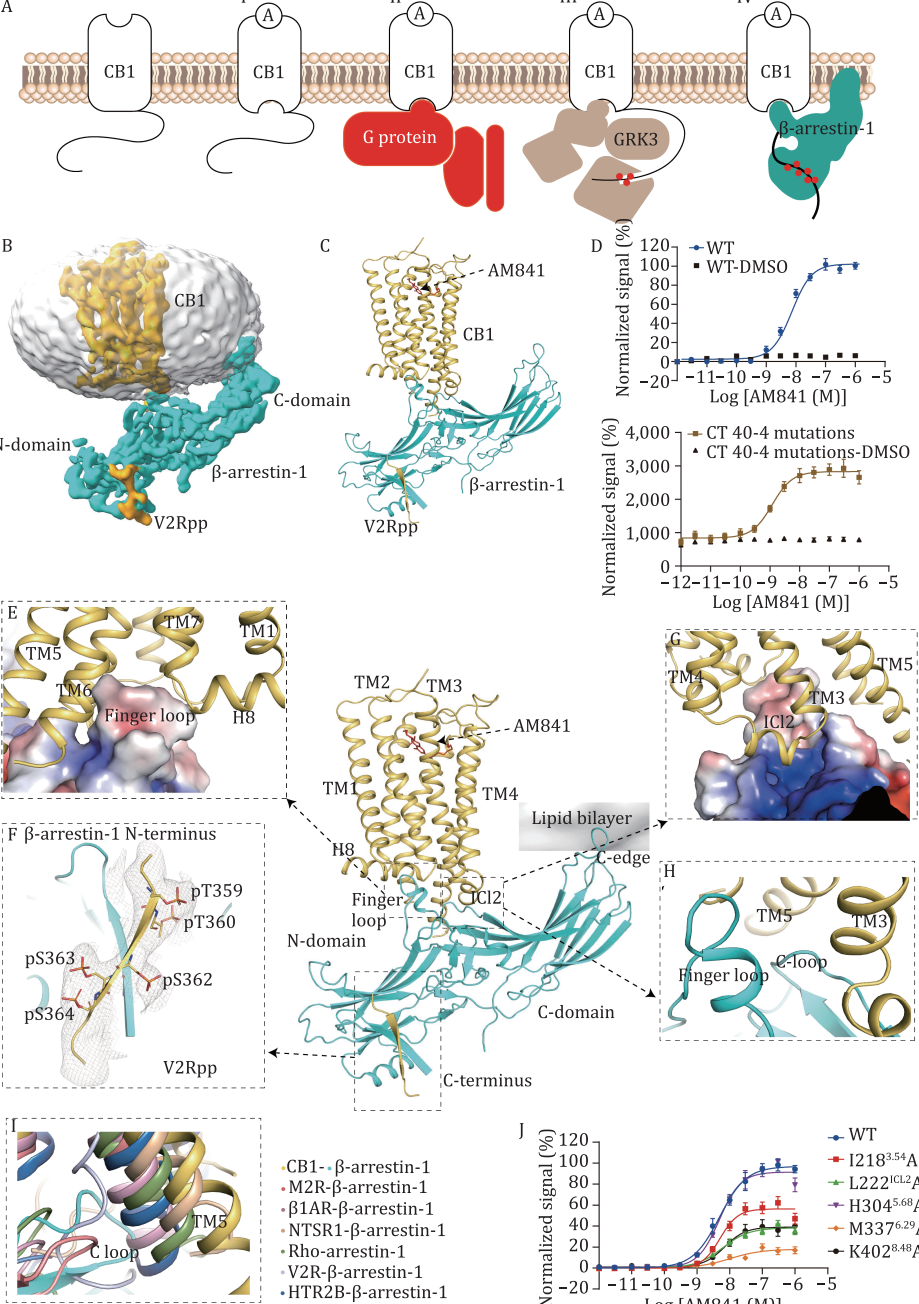
**Cryo-EM structure of CB1-βarr1 complex and interaction mode.** (A) Agonist (A)-induced conformational changes in CB1 (I) lead to heterotrimeric G protein activation (II) and subsequent GRK3-mediated CB1 phosphorylation (red circles) (III). βarr1 then binds to the transmembrane (TM) bundle of phosphorylated CB1 (IV). (B) Cryo-EM density map of phosphorylated CB1_c-V2R_ with V2Rpp (yellow) in complex with AM841 and βarr1. (C) Overall structure of the AM841-CB1_c-V2R_–βarr1 complex. (D) AM841 activated WT CB1 and the construct for structure determination (CT40-4 mutations: CB1 C-terminal truncation of 40 residues 433-472 and 4 mutations (T210^3.46^I, E273^5.37^K, T283^5.47^V, and R340^6.32^E)) in Tango assay. (E) Zoom-in view of the βarr1 finger loop (as electrostatic surface) inserting into the CB1 TM bundle. (F) Zoom-in view of βarr1 N-domain bound to phosphorylated V2R tail fused in CB1 C-terminus. (G) Zoom-in view of the interaction between ICL2 of CB1 and βarr1. (H) Zoom-in view of finger loop and C-loop binding in CB1. (I) A comparison of insertion angles of C-loops in the different GPCR–arrestin complex structures, M2R-β-arrestin-1 (PDB:6U1N), β_1_AR-β-arrestin-1 (PDB: 6TKO), NTSR1-β-arrestin-1 (PDB: 6UP7), Rho-arrestin-1 (PDB: 4ZWJ), V2R-β-arrestin-1 (PDB: 7R0C), HTR2B-β-arrestin-1 (PDB: 7SRS). (J) AM841 induced activation of WT and mutants of CB1 in Tango assay.

Stabilizing the GPCR-β-arrestin complex was challenging due to the requirement for precise receptor phosphorylation and the weak interaction between β-arrestin and receptor. GRK3, co-expressed with βarr1, was reported to induce CB1 desensitization (Jin et al., 1999) (Fig. 1A). To improve the expression and stability of CB1, four mutations, T210^3.46^I, E273^5.37^K, T283^5.47^V, and R340^6.32^E, were introduced to the wild type (WT) receptor. Additionally, to further enhance the phosphorylation level and improve the binding affinity of βarr1 to the receptor, the residues 433–472 of CB1’s C-terminus were truncated and replaced with the C-terminus of vasopressin-2-receptor (V2R), which is referred to as CB1_c-V2R_ (Fig. S1A). Our Tango assay results showed the modified that construct increased the efficacy of AM841 on activating CB1 (Fig. 1D; Table S3). To reconstitute a functional and stable CB1-βarr1 complex, an optimized procedure was performed using different GRKs (GRK1-7) and agonists, co-expressing CB1_c-V2R_ and βarr1 in *Sf*9 cells. The results revealed that GRK3 is better suited for CB1 phosphorylation and shows an increased ability to recruit βarr1. In addition, we optimized the co-expression of CB1_c-V2R_, GRK3, βarr1, along with scFv30 and 1 µmol/L AM841 in *Sf*9 cells. Eventually, a relatively more stable complex was achieved with the AM841-bound CB1_c-V2R_-βarr1 in the presence of scFv30 (Fig. S1B–D). After multiple rounds of 2D and 3D classification, and subsequent 3D refinement focussed on the receptor and its interface with βarr1, a 3.6 Å resolution map yielded relatively well-resolved features for building the complex model (Fig. 1B-C, S2 and Table S1). The density for βarr1 was notably well-defined, while the densities for CB1 and the interaction interface were relatively weak, suggesting that the complex exhibits a high degree of dynamic behaviour. However, the density for the five phosphorylation sites of C-terminus of V2R in the EM map was clear, indicating the homogeneous phosphorylation by GRK3 during co-expression.

The CB_c-V2R_-βarr1 structure reveals a complex and multimodal interaction network between receptor and βarr1. The transmembrane core and intracellular loops ICL2 and ICL3 from CB1 might be involved in the interactions with βarr1. Specifically, the c-terminal tail of V2R binds the N-lobe groove of βarr1, which contains basic residues known to interact with phosphorylated receptor residues (Lee et al., 2020; Staus et al., 2020; Bous et al.,2022). Additionally, the finger loop of βarr1 engages the 7 transmembrane (7TM) core with highly dynamic features (Fig. S3), a crucial step for arrestin coupling. The βarr1 C-edge contacts with the detergent micelle, resulting in a tilt of βarr1 relative to the receptor (Fig. 1B).

Comparing the structures of AM841-bound CB1_c-V2R_-βarr1 and AM841-bound CB1-G_i_ complexes, we observe almost identical binding poses for AM841. Furthermore, the receptor’s 7TM bundle adopts a similar active conformation characterized by the opening of the cytoplasmic part of TM6 (Fig. S4A). Notably, the cytoplasmic end of TM5 in the CB1_c-V2R_-βarr1 structure exhibits an extra outward shift of ~5.2 Å, likely required to accommodate βarr1 (Fig. S4B–D). The βarr1 finger loop binds slightly lower to the receptor core compared to the α5 helix of Gα_i_, and the interaction interface with the receptor is smaller (the interface area between CB1 and βarr1 is 2227.2 Å^2^, while it is 2481.8 Å^2^ for the CB1-G_i_ complex) (Figs. S3 and S5B; Table S2). This difference may explain the conformational flexibility of βarr1-receptor interaction compared to that of G protein (Hilger et al., 2018).

In the CB1_c-V2R_-βarr1 structure, βarr1 engages CB1 in an orientation that differs from that in the M2R-βarr1 or β_1_AR-βarr1 complex, V2R-βarr1 or HTR2B-βarr1, and NTSR1-βarr1 structure (Fig. S6A and S6B). This observation provides further support for the hypothesis that arrestin may adopt distinct, receptor-specific orientations (Hilger et al., 2018).

βarr1 interacts with CB1_c-V2R_ through three main interfaces: the phosphorylated V2R c-terminal tail, the 7TM core, and ICL2 (Fig. 1E–H). The interaction between the phosphopeptide and βarr1 in our structure is nearly identical to that in the V2Rpp-βarr1-Fab30 and M2R-βarr1 structures. The peptide binds to a positively charged crevice in the N-domain, which destabilizes the polar core of arrestin and causes the gate loop to move toward the N-domain (Figs. 1F and S7). The βarr1 finger loop inserts into the receptor 7TM bundle and forms hydrophobic and electrostatic interactions with residues from the cytoplasmic parts of TM3, TM6, as well as helix 8 and ICL2 (Fig. 1E). In addition, alanine mutations of residues involved in the interaction interface, such as I218^3.54^, L222^ICL2^, M337^6.29^, and K402^8.48^, decrease the β-arrestin recruitment of CB1 in Tango assay (Fig. 1J; Table S3). Compared to the solved structures of NTSR1-βarr1 (Huang et al., 2020), M2R-βarr1 (Staus et al., 2020), β_1_AR-βarr1 (Lee et al., 2020), V2R-βarr1 (Bous et al., 2022), and HTR2B-βarr1 (Cao et al., 2022), the binding position of βarr1 finger loop relative to the receptor core in CB1 is more similar to that in NTSR1, M2R, and HTR2B, while it inserts much deeper in the β_1_AR structure and much lower in the V2R structure (Fig. S6C). Notably, TM5 of CB1 shows the largest outward rotation among the six complex structures (Fig. S5A and S5B). Consistently, the density map of βarr1 finger loop is relatively weak in the CB1_c-V2R_-βarr1 structure and 3D variability analysis shows the flexibility of the finger loop in βarr1 (Fig. S3).

Interestingly, the extended TM5 in CB1 appears to form additional interactions with the C-loop of βarr1 (Fig. 1H). Here, the C-loop packs closer to TM5 and the βarr1 finger loop, forming hydrophobic interactions, which is different from other complex structures (Fig. 1I). The C-loop of βarr1 shows interactions with ICL2 in NTSR1-βarr1, β_1_AR-βarr1, and HTR2B-βarr1 structures, but shows no obvious interactions in the V2R-βarr1 and M2R-βarr1 structures (Bous et al., 2022; Cao et al., 2022). This observation supports the potential role of C-loop in βarr1 recruitment. In the CB1_c-V2R_-βarr1 structure, ICL2 forms a short helix and is located in the cavity formed by the finger loop, C-loop, middle-loop, and lariat-loop (Fig. 1G).

Previous studies have shown that the interaction between arrestin’s C-edge and membrane lipids is important for stabilizing the active βarr1 conformation, which may also modulate the receptor desensitization and internalization (Lally et al., 2017). Interestingly, in the CB1_c-V2R_-βarr1 structure, both C-edge loops (197-loop and 344-loop) (Lally et al., 2017) interact with the membrane and insert deeper into the micelle compared to other solved GPCR-arrestin structures. This unique interaction likely contributes to the specific orientation of βarr1 in CB1 (Fig. S6A).

In NTSR1-βarr1 structure, a phospholipid (PIP2) was proposed to bind between the membrane surface of TMs 1 and 4 and the top of the arrestin C-lobe (Huang et al., 2020), playing an important role in stabilizing NTSR1-βarr1 complex. However, in the CB1_c-V2R_-βarr1 structure, the density for PIP2 is absent, and the proposed key residues (K233, R236, and K250) in βarr1, important for PIP2 binding, show similar sidechain orientations despite being slightly further away from the membrane plane (Fig. S5C).

The mechanism of biased signalling of CB1 is still being explored, while most agonists including AM841 are reported to activate both G protein and β-arrestin pathways. Here, we obtained both AM841-activated CB1-G_i_ and CB1-βarr1 complex structures. The overall structures of the receptors in the two complexes are quite similar, with a root mean square deviation value for Cα atoms of 1.26 Å, but some noteworthy differences were observed (Fig. S4B–D). Though the density for the receptor in CB1-βarr1 complex is low, the 7TM bundles could be clearly modelled.

In CB1_c-V2R_-βarr1 structure, TM1 and TM4 show conformational changes compared with that in CB1-G_i_ complex structure (Fig. S4B–D). As mentioned earlier, the major difference in βarr1-coupled CB1 is the outward movement of the cytoplasmic part of TM5, while TM6 adopts a similar conformation compared to that in the G_i_-bound CB1 structure (Fig. S4B). These observations provide a valuable model describing the changes that occur in CB1 between the G protein-coupled state to the arrestin-coupled state.

In this study, we used a new approach to capture the agonist AM841-activated CB1 in complex with β-arrestin-1 through co-expression with GRK3 in insect cells. This method allowed us to capture a core-engagement state of CB1-βarr1 complex, despite yielding slightly lower density maps compared to those obtained using the crosslinking method. Importantly, we successfully utilized GRK3 for GPCR phosphorylation and β-arrestin recruitment. Furthermore, the diversity in arrestin engagement with CB1 observed in this study supports the notion that receptor coupling and membrane anchoring contribute to the plasticity of receptor–arrestin interactions. Given the wide distribution of CB1 receptors in the human body, it is important to dissect the molecular mechanisms to design organ and pathway-specific ligands, including phytocannabinoids, endocannabinoids, and synthetic ligands, for disease-specific applications. This study provides additional structural templates of CB1 to guide the design of biased ligands for this important target.

## Supplementary Material

pwad055_suppl_Supplementary_Figures_S1-S6_Tables_S1-S3

## Footnotes

We thank Dr. Alexandros Makriyannis for providing agonist AM841. We thank the Shanghai Municipal Government and ShanghaiTech University for financial support. The cryo-EM data were collected at the Bio-Electron Microscopy Facility, ShanghaiTech University, with the assistance of Z.H.Z. We also thank the staff members of Assay, Cell Expression, Cloning, and Purification Core Facilities of iHuman Institute for their support. Coordinates and structure factors have been deposited in the Protein Data Bank for AM841-CB1_c-V2R_-βarr1 (PDB: 8WRZ). This article does not contain any studies with human or animal subjects performed by any of the authors. The authors consent for participation and for publication.

Conceptualization—T.H. and Z.-J.L.; Methodology—Y.W., L.W., T.W., J.L., Z.F., Y.Y., L.J., N.C., Q.S., F.L., and Q.T.; Validation—L.W.; Formal Analysis—Y.W., L.W., T.W., T.H., and Z.-J.L.; Investigation—T.H. and Z.-J.L.; Writing-Original Draft—T.H., Y.W., and Z.-J.L.; Writing-Reviewing, & Editing—T.H. and Z.-J.L.; Visualization—T.H., Y.W., and T.W.; and Supervision—T.H. and Z.-J.L.

The authors declared no conflict interests.

This work was supported by the National Key Research and Development Program of China grant 2022YFA1302903 (T.H.), the National Natural Science Foundation of China grant 91953202 (Z.-J.L.), the CAS Strategic Priority Research Program XDB37030104 (Z.-J.L.), the National Science Fund for Distinguished Young Scholars 32022038 (T.H.), the National Natural Science Foundation of China grants 32230026 (Z.-J.L.) and 32271262 (T.H.), and Shanghai Frontiers Science Center for Bomacromolecules and Precision Medicine.
