# Supplementary material for: Cryo-EM structure of cannabinoid receptor CB1-β-arrestin complex
Source: Protein Cell. 2023 Dec 15;15(3):230–4. doi: 10.1093/procel/pwad055 (PMC10903984; doi:10.1093/procel/pwad055)
Supplement: pwad055_suppl_Supplementary_Figures_S1-S6_Tables_S1-S3 [file pwad055_suppl_supplementary_figures_s1-s6_tables_s1-s3.pdf]

Supplementary Materials

Materials and Methods

Constructs of CB1, GRK3, βarr1, and scFv30

The CB1 construct was similar to the construct that used in the AM841-CB1-G_i complex determination (Hua et al., 2020). Briefly, wild type human CB1 was subcloned into a modified pFastBac1 vector, which contains a haemagglutinin (HA) signal sequence, a FLAG tag and 10 × His tag at the N terminus. In order to improve the protein expression, the construct was further modified by the truncations of the CB1 residues 1-70, and 433-472, and the BRIL (PDB: 1M6T, MW 10.9kDa) fusion protein was fused to the N terminus, and mutations of $T210^{3.46}I$, $E273^{5.37}K$, $T283^{5.47}V$ and $R340^{6.32}E$ were introduced. In order to promote CB1-βarr1 combination, a V2R tail (ARGRTPPSLGPQDESCTTASSSLAKD) was fused to the C terminus of CB1. This construct was referred as $CB1_{c\text{-}V2R}$.

Untagged full length human GRK3, untagged human βarr1 (1-393) and untagged scFv30 (Shukla et al., 2014) were cloned in pFastBac1 vector, individually.

$CB1_{c\text{-}V2R}$-βarr1 Complex Expression, Formation, and Purification

To form complex, we also used the co-expression method similar to $β_2$AR–βarr1 complex (Shukla et al., 2014). Differently, we co-expressed scFv30 (Shukla et al., 2013) with $CB1_{c\text{-}V2R}$, GRK3 and βarr1, which significantly improve the complex stability. Briefly, using Bac-to-Bac Baculovirus Expression System (Invitrogen), *Sf*9 cells were infected at a cell density of 2-2.5 × 10^6 cells per ml with four separate virus preparations for $CB1_{c\text{-}V2R}$, GRK3, βarr1 and scFv30 at a ratio of 1:1:1:1. The infected cells were cultured at 27 °C for 66 h, then cells were stimulated for 1 h with 1 μM AM841 to stimulate receptor phosphorylation and complex formation. Cells were collected by centrifugation and stored at -80 ℃ for future use.

The cell pellets from 1 L $CB1_{c\text{-}V2R}$-βarr1-scFv30 co-expression culture were thawed and lysed in the hypotonic buffer (10 mM HEPES pH 7.5, 10 mM $MgCl_2$, 20 mM KCl) with EDTA-free complete protease inhibitor cocktail tablets (Roche) (Zhang et al., 2020). $CB1_{c\text{-}V2R}$-βarr1-scFv30 complex was formed in membranes by addition of 10 μM AM841. The lysate was incubated for overnight at 4 ℃ and discard the supernatant by centrifugation at 35,000 rpm for 35 min. The complex from membranes was solubilized in 1 × solubilization buffer (20 mM HEPES pH 7.5, 100 mM NaCl, 0.75% (w/v) lauryl maltose neopentyl glycol (LMNG, Anatrace), 0.15% (w/v) cholesterol hemisucinate (CHS, Sigma-Aldrich)), with 10 μM AM841 at 4 °C for 3 h. The supernatant was isolated by ultracentrifugation, and then incubated with TALON IMAC resin (Clontech) and 20 mM

imidazole overnight at 4 ℃. The resin was washed with 20 CV (column volumes) with washing buffer (25 mM HEPES pH 7.5, 100 mM NaCl, 10% (v/v) glycerol, 0.1% (w/v) LMNG, 0.02% (w/v) CHS, 30 mM imidazole and 5 μM AM841). The protein was eluted using 4 CV of elution buffer (25 mM HEPES pH 7.5, 100 mM NaCl, 10% (v/v) glycerol, 0.01% (w/v) LMNG, 0.002% (w/v) CHS, 250 mM imidazole and 25 μM AM841). The purified $CB1_{c\text{-}V2R}$-βarr1 complex was concentrated, then injected onto a Superdex200 10/300 GL column (GE Healthcare) equilibrated in the FPLC buffer (20 mM HEPES pH 7.5, 100 mM NaCl, 0.00075% (w/v) LMNG, 0.00025% GDN, 0.0001% (w/v) CHS). The complex peak fractions were collected and concentrated individually to 1.5 mg/mL for electron microscopy experiments.

Cryo-EM Sample Preparation and Image Acquisition

Three microliters of purified $CB1_{c\text{-}V2R}$-βarr1 complexes was applied to a glow-discharged holey carbon grid, and subsequently vitrified using a Vitrobot Mark IV (Thermo Fisher Scientific). The chamber of Vitrobot was set to 100% humidity at 4 ℃. The sample was blotted for 3 s with blot force 2. Cryo-EM images were collected on a Titan Krios microscope operated at 300 kV equipped with a Gatan Quantum energy filter, with a slit width of 20 eV, a Gatan K3 summit direct electron camera (Gatan). Movies were taken in EFTEM nanoprobe mode, with 70 μm C2 aperture, at a calibrated magnification of 130,000 corresponding to a magnified pixel size of 0.832 Å on the specimen level. Each movie stack was recorded for 2.0 s with 0.05 s exposure per frame, a dose rate of 20 electrons per $Å^2$ s^{-1} and a total dose of 60 electrons per $Å^2$. Data was acquired using SerialEM software(Mastronarde, 2005) with a defocus value in the range of −1.0 to −2.0 μm.

Image Processing and 3D Reconstruction

For $CB1_{c\text{-}V2R}$-βarr1 complex, a total of 25,847 images were collected and analyzed with cryoSPARC v3.2 (Punjani et al., 2017). Beam-induced motion correction was performed using patch motion correction. Contrast Transfer function (CTF) parameters for each dose-weighted micrograph were estimated by patch CTF estimation in cryoSPARC. To perform particle using Topaz, a model firstly was trained using the Topaz Train job with 500 images. A total of 8,451,168 particles were autopicked, and was used to do two cycles of 2D classification. Then a total of 1,904,015 particles were selected for 3D classification in cryoSPARC. 42% ($CB1_{c\text{-}V2R}$-βarr1) of particles was selected for further 3D classifications. The final dataset of 130,058 particle projections from the best class was further applied for final homogenous refinement in cryoSPARC, a density map was obtained with nominal

resolution of 3.6 Å (determined by gold standard Fourier shell correlation (FSC) using the 0.143 criterion).

Model Building and Refinement

For the $CB1_{c\text{-}V2R}$-βarr1 complex, CB1-AM841 in CB1-G_i-scFv16 complex structure (PDB code 6KPG) (Hua et al., 2020) and βarr1-scFv30 in M2R-βarr1 complex structure (PDB code 6UIN) (Staus et al., 2020) were used as the starting models for model building and refinement against the electron density map. The cryo-EM model was docked into the electron microscopy density map using Chimera (Pettersen et al., 2004), followed by iterative manual adjustment and rebuilding in Coot (Emsley et al., 2010) and phenix.real_space_refine in PHENIX (Adams et al., 2010). The model statistics were validated using MolProbity (Chen et al., 2010). Structural figures were prepared in Chimera and PyMOL (http://www.pymol.org). The final refinement statistics are provided in Supplementary Table 1. The extent to which any model was overfitted during refinement was measured by refining the final model against one of the half-maps and by comparing the resulting map versus model FSC curves with the two half-maps and full model.

β-arrestin 2 recruitment assay

The CB1 mediated β-arrestin 2 translocation activity was measured by a PRESTO-Tango assay kit (a gift from Bryan Roth), following the manufacturer's protocol (Addgene kit #1000000068). HTLA cells were plated at 1.5×10^6 to 2.0×10^6 cells per 60-mm cell culture dish. The cells were transfected with CB1-Tango constructs overnight using the calcium chloride method when they reached a confluency of 60-70%. Subsequently, the cells were collected and seeded into poly-l-Lys (PLL)-coated 384-well plates, with 15,000 cells in a volume of 40 μl per well, using DMEM supplemented with 1% dialysed FBS. After 6 h, 5× compound stimulation solutions were prepared in the medium (DMEM and 1% dialysed FBS), and 10 μl of each solution was added to the corresponding wells. One day after incubation, the growth medium and compound solutions were carefully removed from the wells and immediately replaced with 20 μl per well of Britelite solution diluted 20-fold in assay buffer (1×HBSS and 20 mM HEPES, pH7.4). After incubation for 15-20 min at room temperature, luminescence was measured using an Envision luminescence counter.

References

Adams, P.D., Afonine, P.V., Bunkoczi, G., Chen, V.B., Davis, I.W., Echols, N., Headd, J.J., Hung, L.W., Kapral, G.J., Grosse-Kunstleve, R.W., *et al.* (2010). PHENIX: a comprehensive

Python-based system for macromolecular structure solution. Acta Crystallogr D Biol Crystallogr 66, 213-221.

Chen, V.B., Arendall, W.B., 3rd, Headd, J.J., Keedy, D.A., Immormino, R.M., Kapral, G.J., Murray, L.W., Richardson, J.S., and Richardson, D.C. (2010). MolProbity: all-atom structure validation for macromolecular crystallography. Acta Crystallogr D Biol Crystallogr 66, 12-21.

Emsley, P., Lohkamp, B., Scott, W.G., and Cowtan, K. (2010). Features and development of Coot. Acta Crystallogr D Biol Crystallogr 66, 486-501.

Hua, T., Li, X., Wu, L., Iliopoulos-Tsoutsouvas, C., Wang, Y., Wu, M., Shen, L., Brust, C.A., Nikas, S.P., Song, F., *et al.* (2020). Activation and Signaling Mechanism Revealed by Cannabinoid Receptor-Gi Complex Structures. Cell 180, 655-665 e618.

Mastronarde, D.N. (2005). Automated electron microscope tomography using robust prediction of specimen movements. J Struct Biol 152, 36-51.

Pettersen, E.F., Goddard, T.D., Huang, C.C., Couch, G.S., Greenblatt, D.M., Meng, E.C., and Ferrin, T.E. (2004). UCSF Chimera--a visualization system for exploratory research and analysis. J Comput Chem 25, 1605-1612.

Punjani, A., Rubinstein, J.L., Fleet, D.J., and Brubaker, M.A. (2017). cryoSPARC: algorithms for rapid unsupervised cryo-EM structure determination. Nat Methods 14, 290-296.

Shukla, A.K., Manglik, A., Kruse, A.C., Xiao, K., Reis, R.I., Tseng, W.C., Staus, D.P., Hilger, D., Uysal, S., Huang, L.Y., *et al.* (2013). Structure of active beta-arrestin-1 bound to a G-protein-coupled receptor phosphopeptide. Nature 497, 137-141.

Shukla, A.K., Westfield, G.H., Xiao, K., Reis, R.I., Huang, L.Y., Tripathi-Shukla, P., Qian, J., Li, S., Blanc, A., Oleskie, A.N., *et al.* (2014). Visualization of arrestin recruitment by a G-protein-coupled receptor. Nature 512, 218-222.

Staus, D.P., Hu, H., Robertson, M.J., Kleinhenz, A.L.W., Wingler, L.M., Capel, W.D., Latorraca, N.R., Lefkowitz, R.J., and Skiniotis, G. (2020). Structure of the M2 muscarinic receptor-beta-arrestin complex in a lipid nanodisc. Nature 579, 297-302.

Zhang, J., Qu, L., Wu, L., Tang, X., Luo, F., Xu, W., Xu, Y., Liu, Z.-J., and Hua, T. (2020). Structural insights into the activation initiation of full-length mGlu1. Protein & Cell 12, 662-667.

Supplementary Figure 1 Sample preparation for AM841-CB1$_{c\text{-}V2R}$-βarr1 complex. (A) The construct design for AM841-CB1$_{c\text{-}V2R}$-βarr1-scFv30 complex formation *in situ*. (B) The expression and purification procedure of AM841-CB1$_{c\text{-}V2R}$-βarr1-scFv30 complex. (C) The Superdex200 size-exclusion chromatography elution profiles of the purified AM841-CB1$_{c\text{-}V2R}$-βarr1-scFv30 complex. (D) The SDS-PAGE and western blot analysis of AM841-CB1$_{c\text{-}V2R}$-βarr1-scFv30 complex sample.

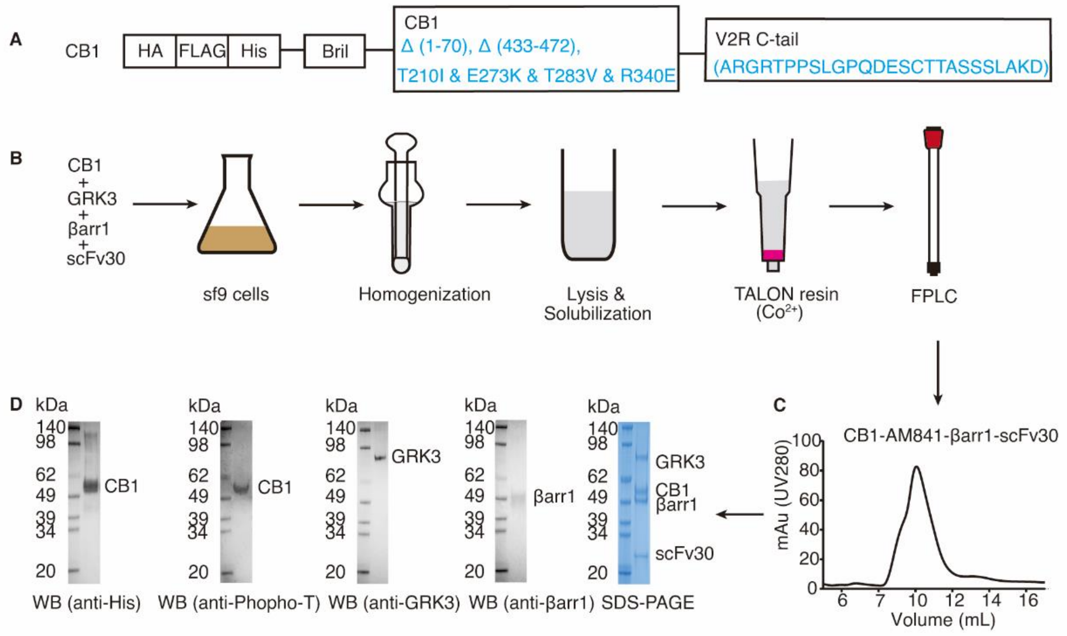

Supplementary Figure 2 Cryo-EM data processing of AM841-CB1$_{c\text{-}V2R}$-βarr1 complex. (A) Representative cryo-EM image of AM841-CB1$_{c\text{-}V2R}$-βarr1-scFv30 complex (scale bar: 50 nm). (B) Representative 2D classification showing distinct structural features of each component. Scale bar: 5 nm. (C) Workflow of cryo-EM data processing for AM841-CB1$_{c\text{-}V2R}$-βarr1-scFv30 complex. (D-E) Cryo-EM maps are colored by local resolution (Å). 'Gold-standard' FSC curve of the AM841-CB1$_{c\text{-}V2R}$-βarr1-scFv30, indicating the resolution at the FSC=0.143 is 3.6 Å. (F) Density maps for loops in βarr1 which have interactions with CB1.

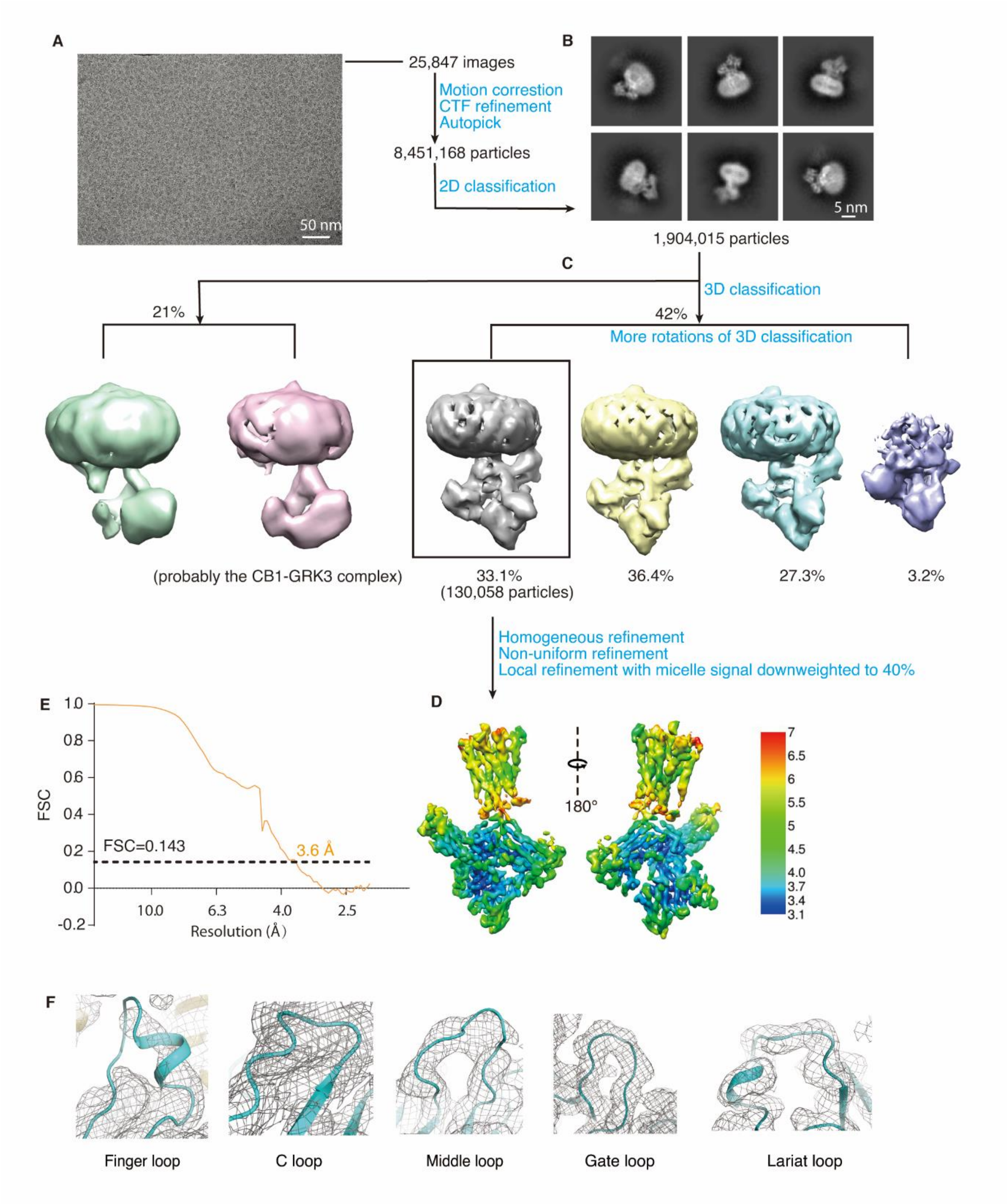

Supplementary Figure 3 Flexibility analysis of the finger loop in βarr1 in coupling with CB1. Frame 0, 6, 10, 12, 14, 19 are representative captures of the finger loop at different states. Data were obtained using "3D Variability Analysis" in CryoSPARC.

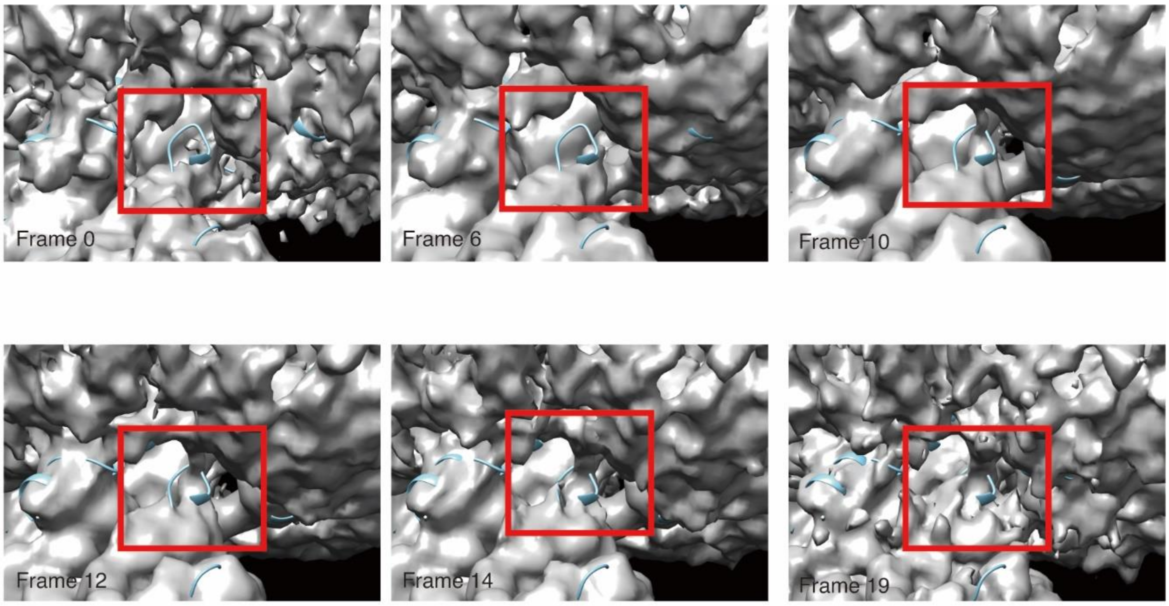
Frame 0
Frame 6
Frame 10
Frame 12
Frame 14
Frame 19

Supplementary Figure 4 Structural comparison of CB1 in complex with G_i and βarr1 complexes. (A) Structural comparison of the AM841-$CB1_{c\text{-}V2R}$-βarr1 and AM841-CB1-G_i (PDB: 6KPG) complexes. Inset shows enlargement of the βarr1 finger loop and the G_i α5 helix. (B-D) Superposition of the receptors coupled to G_i (blue) and βarr1 (yellow), and antagonist AM6538 bound CB1 (grey) structures when aligned the receptor, AM841-CB1-Gi (PDB: 6KPG), AM6538-CB1 (PDB: 5TGZ).

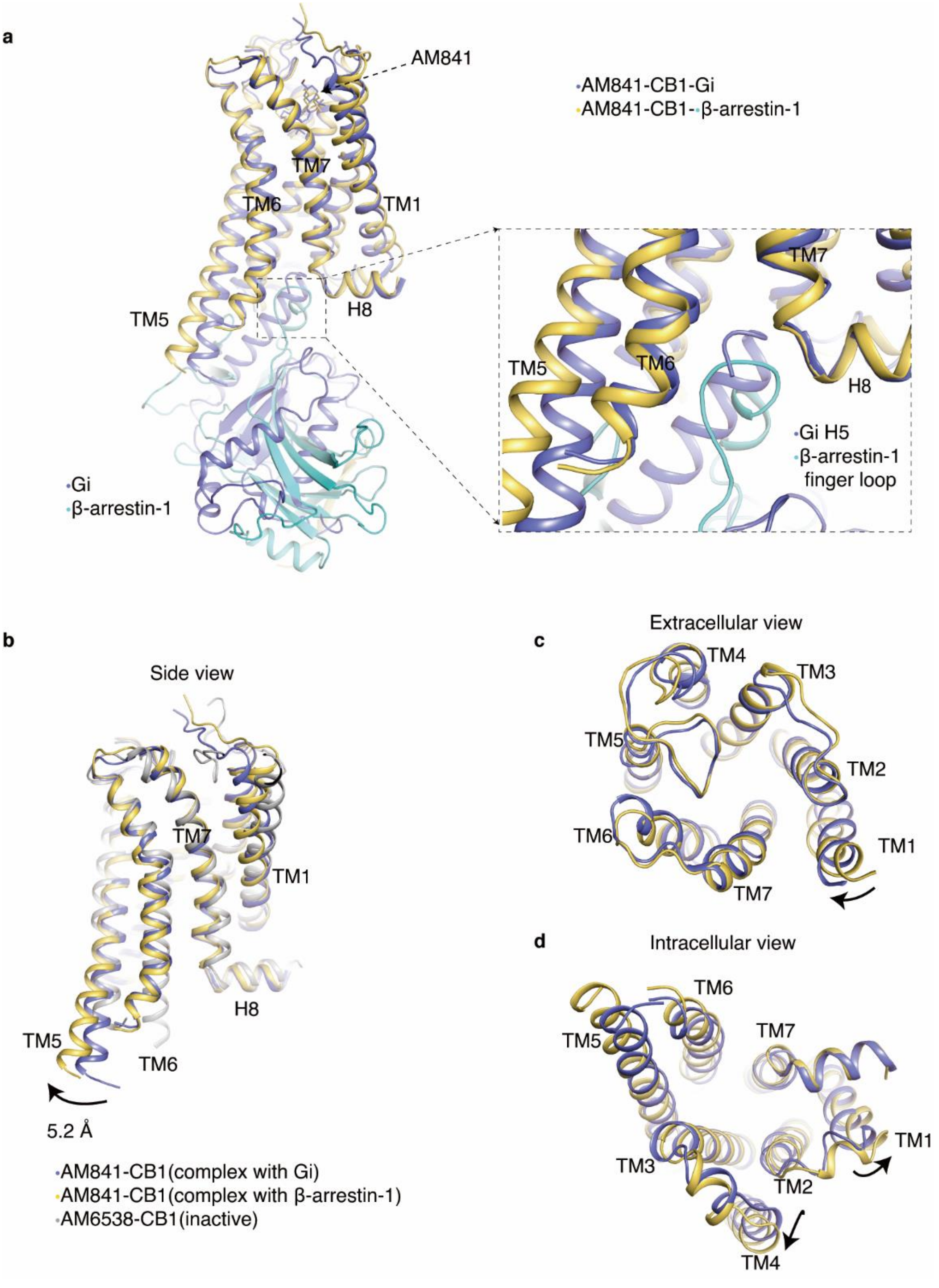

Supplementary Figure 5 Comparison of the TM5 movements and arrestin interaction interfaces in different GPCR-arrestin/G protein complex structures. (A) Comparison of TM5 movements in different GPCR-arrestin/G protein complex structures. (B) Comparison of the interaction interfaces in different GPCR-arrestin complex structures. (C) PIP2 binding region in NTSR1-βarr1 and its comparison with βarr1 in CB1-βarr1 structure. Structures used are CB1-β-arrestin-1, CB1-Gi (PDB: 6KPG), M2R-β-arrestin-1 (PDB: 6U1N), M2R-Go (PDB: 6OIK), NTSR1-β-arrestin-1 (PDB: 6UP7), NTSR1-Gi1 (PDB: 6OS9), HTR2B-β-arrestin-1 (PDB: 7SRS), HTR2B-miniGq (PDB: 7SRR), V2R-β-arrestin-1 (PDB: 7R0C), V2R-Gs (PDB: 7BB7), β1AR-β-arrestin-1 (PDB: 6TKO), β1AR-Gs (PDB: 7JJO).

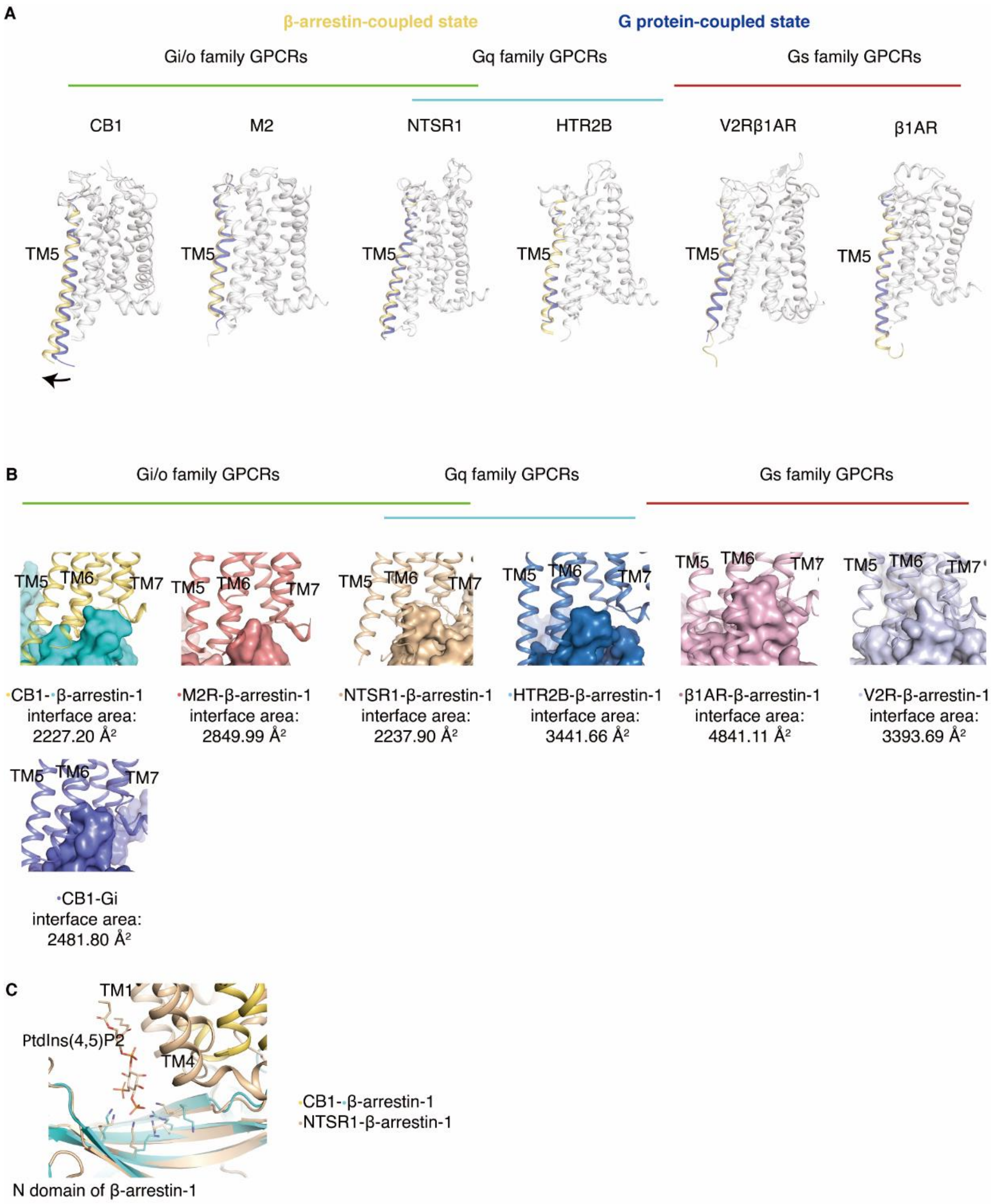

Supplementary Figure 6 Comparison of receptor/arrestin interfaces in different GPCR-arrestin complexes. (A) A comparison of the CB1-βarr1 coupling mode with the M2-βarr1 (PDB: 6OIK), β1AR-βarr1 (PDB: 6TKO), Rho-arrestin 1 (PDB: 4ZWJ), HTR2B-βarr1 (PDB: 7SRS), V2R-βarr1 (PDB: 7R0C) and NTSR1-βarr1 (PDB: 6UP7). (B) Bottom view of the orientation of arrestin relative to the receptor in different complexes when aligned on the receptor 7TM bundle. (C) A comparison of finger loops in the different GPCR-arrestin complex structures, colours as in (B).

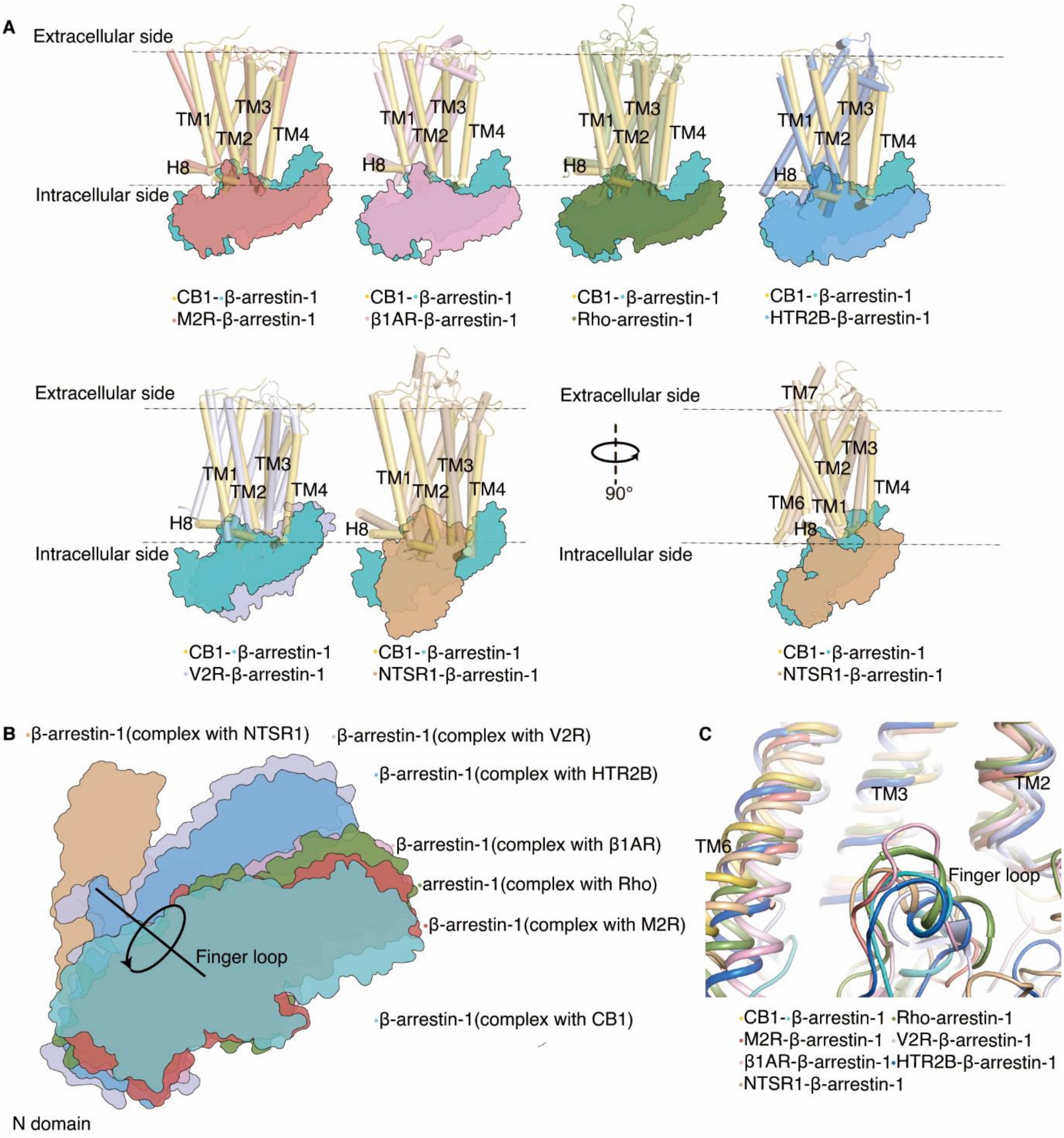

Supplementary Figure 7 Comparison of arrestin in inactive state and GPCR-arrestin complex structures. (A) Structural comparison of inactive state of βarr1 (PDB: 1G4M) and βarr1 in AM841-CB1$_{c\text{-}V2R}$-βarr1 complex. (B) Structural comparison of active state of βarr1 (PDB: 4JQI) and βarr1 in AM841-CB1$_{c\text{-}V2R}$-βarr1 complex. (C) Structural comparison of βarr1 in AM841-CB1$_{c\text{-}V2R}$-βarr1 and M2R-βarr1 (PDB: 6OIK) complexes. **(D)** The conformational changes of N domains of βarr1 in different states and complex structures. The red arrows indicate the conformational changes.

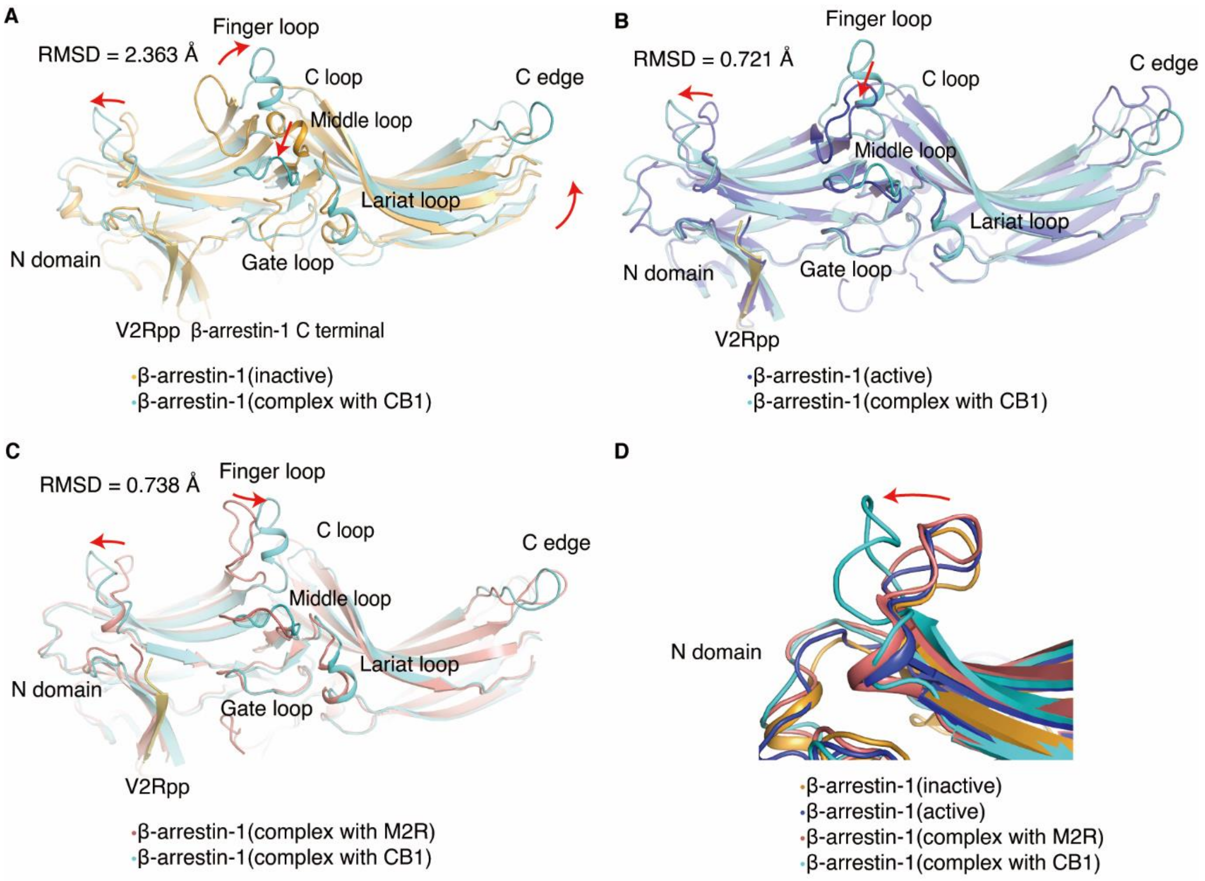

Supplementary Table 1 Cryo-EM Data Collection, Model Refinement and Validation

	CB1-AM841-βarr1-scFv30 (EMDB-33314) (PDB-8WRZ)
**Data collection and processing**	
Magnification	130,000
Voltage (kV)	300
Electron exposure ($e^-/Å^2$)	60
Defocus range (μm)	-1.0~-2.0
Pixel size (Å)	0.832
Symmetry imposed	C1
Initial particle images (no.)	8,451,168
Final particle images (no.)	130,058
Map resolution (Å)	3.6
FSC threshold	0.143
Map resolution range (Å)	3.1-7.0
**Refinement**	
Initial model used (PDB code)	6KPG, 6U1N
Model resolution (Å)	3.6
FSC threshold	0.143
Map sharpening *B* factor ($Å^2$)	138.7
Model composition	
Non-hydrogen atoms	6853
Protein residues	874
Ligands	31
B factors ($Å^2$)	
Protein	128.6
Ligand	154.3
R.m.s. deviations	
Bond lengths (Å)	0.004
Bond angles (°)	0.8
Validation	
MolProbity score	2.14
Clashscore	17.65
Poor rotamers (%)	0.13
Ramachandran plot	
Favored (%)	94.26
Allowed (%)	5.74
Disallowed (%)	0

Supplementary Table 2 The interaction interface area of receptors with βarr1 and Gα subunit, calculated in Pymol

	HTR2B-βarr1	V2R-βarr1	M2R-βarr1	NTSR1-βarr1	β1AR-βarr1	CB1-βarr1
PDB	7SRS	7R0C	6U1N	6UP7	6TKO	8WRZ
GPCR- βarr1 Interface Area (Å^2)	3441.66	3393.69	2849.99	2237.90	4841.11	2227.20

	HTR2B-miniGq	V2R-Gs	M2R-Go	NTSR1-Gi1	β1AR-Gs	CB1-Gi
PDB	7SRR	7BB7	6OIK	6OS9	7JJO	6KPG
GPCR-Gα Interface Area (Å^2)	2259.43	2618.86	1756.01	2535.37	2329.12	2481.80

Supplementary Table 3 The Tango assay of wild-type (WT) and mutant CB1

	pEC_{50}	E_{max} (%)
WT	8.23 ± 0.19	100 ± 0.00
I218$^{3.54}$A	8.28 ± 0.07	56.64 ± 8.91**
L222^{ICL2}A	8.12 ± 0.14	40.28 ± 5.38***
H304$^{5.68}$A	8.31 ± 0.02	91.57 ± 4.90
M337$^{6.29}$A	7.99 ± 0.17	18.23 ± 3.28****
K402$^{8.48}$A	8.17 ± 0.10	39.20 ± 12.56***
	pEC_{50}	E_{max} (%)
WT	8.29 ± 0.07	100 ± 0.00
CT40-4 mutations	8.94 ± 0.07***	2831 ± 353.4***

Data are shown as mean ± SEM of at least three independent experiments performed in technical replicates. All mutants changed the activity of CB1 significantly analyzed with one-way ANOVA followed by Dunnett's multiple comparisons tests; (ns) p>0.12340, *p<0.0332, **p<0.0021, ***p<0.0002, ****p<0.0001.
